# Physical Self Matters: How the Dual Nature of Body Image Influences Smart Watch Purchase Intention

**DOI:** 10.3389/fpsyg.2022.846491

**Published:** 2022-03-25

**Authors:** Teng Wang, Yongqiang Sun, Shengwu Liao

**Affiliations:** ^1^School of Management, Harbin Institute of Technology, Harbin, China; ^2^School of Information Management, Wuhan University, Wuhan, China; ^3^Southern Hospital of Southern Medical University, Guangzhou, China

**Keywords:** physical self, body image, smart watch, competitive mediation, mobile health

## Abstract

To determine the role of physical self in body-involving consumption, we explore how body image influences purchasing intention toward hybrid products with body-involving features. In this study, we establish the dual nature of body image: specifically, body image influences intention to purchase via the perception of utilitarian value and symbolic value. Further, we find a competitive mediation in which positive body image (PBI) negatively influences purchase intention (direct effect), while PBI is positively related to purchase intention via utilitarian and symbolic value (indirect effect). This indicates that without the mediation testing of the utilitarian-symbolic framework, the positive influence of body image will be “hidden.” Additionally, the mediated effect of symbolic value is moderated by personal innovativeness toward technology (PITT), suggesting that a consumer’s knowledge of wearables enhances the effect of body image. With the introduction of body image, this paper provides a more comprehensive model to analyze purchase intention with regard to digital products with body-involving features.

## Introduction

Mobile health (m-health) emphasizes the role of mobile technology in health promotion (Lupton, 2018). Digital products such as smart phones, smart watches, and smart bracelets enable consumers to record and receive feedback on their health condition, and they further empower consumers in self-care (Wu et al., 2019; Su et al., 2020). International Data Corporation (IDC) has predicted that the wearables market will maintain double-digit growth from 2020 through 2024, with the shipment volume to reach a total of 637.1 million units in 2024.^1^ Thus, the rapid spread of wearables such as smart watches provide a promising platform for m-health service via empowering consumers to perform self-care and self-management.

Recent studies have reached a consensus that digital wearables are categorized as hybrid products, as they involve features of different products (Chuah et al., 2016; Nieroda et al., 2018). For instance, they involve features of both mass fashion (e.g., more affordable fashion) and luxury fashion (e.g., demonstrating higher social status) (Nieroda et al., 2018), have both utilitarian and non-utilitarian aspects (Choi and Kim, 2016), and are both fashion (e.g., are visible to others) and technology (e.g., increase productivity) (Chuah et al., 2016). Health-related wearables have similar hybrid characteristics: on one hand, smart watches have functions including activity tracing, sleep monitoring and heart rate recording, and such functions meet users’ demand for health-related functionality; on the other hand, smart watches are also viewed as fashionable accessories that signal users’ social image and social status (Chuah, 2019). Given these characteristics, digital wearables as hybrid products have unique features that distinguish them from traditional digital devices.

Two reasons drive this research. First, as hybrid products, the body-involving feature of smart watches has been neglected in previous literature. Body-involving products can be defined as products for which consumers make purchase decisions relying on information about their body (Rosa et al., 2006), and these products include cosmetic surgery, weight loss services, fitness services, and fitness accessories. Prior research assumes that consumers make decisions based solely on the perception of the product (e.g., perceived value, quality, and usefulness), ignoring the fact that the perception of one’s own body also relates to the decision making process (Rosa et al., 2006; Gillen and Dunaev, 2017; Yim and Park, 2019). Body-involving features should be taken into consideration and incorporated into a more comprehensive model that will help researchers understand the hybrid feature of digital wearables. Nevertheless, to the best of our knowledge, research on the body dimension of digital wearables is lacking, and our study intends to fill this gap.

Second, despite the role of self-concepts in consumption has been noticed in past works, such as consumption can build extended self (Belk, 1988), with the emergence of digital wearables, the relationship between physical self and consumption has remained unknown. According to multiple self-aspects framework, self-concepts are multiple, specifically, one’s self-concept includes the acknowledgment of roles, identities and social relationships; some selves are more decisive than others (Elster, 1987; McConnell, 2011). Similarly, some research argue that self is multifaceted and hierarchically organized, and one dimension of self has different subareas (e.g., peers and significant others of social self) (Marsh and Shavelson, 1985). Following this stream, self-concepts can be divided into global self and domain-specific self. For example, global self-esteem is one’s attitude toward the self as a totality (Rosenberg et al., 1995), whereas specific esteem, such as academic esteem, reflects one’s agreement with indicators of academic achievement (Marsh, 1990). The global self is associated with psychological wellbeing, and domain-specific self is more relevant to specific behavior (Rosenberg et al., 1995). Physical self involves the perception and evaluation of one’s physical ability and appearance (Shavelson et al., 1976). Previous research has found that the physical self-influences behaviors. For example, according to the self-discrepancy theory, the discrepancy between actual and ideal self generates specific emotions (Higgins, 1987), similar results have been found in physical self-research such as body image discrepancy (Thompson and Gray, 1995), more recent research indicates that patients will experience self-fragmentation, and injured self will alter one’s internal motivation (Sebri et al., 2020), patients’ psychological states are related to their perception of the discrepancy between actual and ideal physical self (Triberti et al., 2019).

Millennials are young consumers born between1980s and 2000s, past research indicate that their consumption motivation are more sophisticated than other groups (Shin et al., 2017), but our knowledge on how physical self-influence millennials’ consumption in digital wearables are still lacking. Therefore, based on the work of Chuah et al. (2016) and Yim and Park (2019), we evaluate the theoretical foundation of body-involving consumption based on self-concepts. Past research has noted the role of self-concepts in consumption behavior, with most focusing on global self-concepts (e.g., self-esteem, self-image, and identity), arguing that symbolic consumption can facilitate realize idealized self (Hogg and Michell, 1996; Banister and Hogg, 2004), and extended self can be built (Belk, 1988). In this study, we consider body image as domain-specific self (physical self) rather than global self to study how body-involving features influence purchase intention. With the introduction of utilitarian (e.g., benefits to health) and symbolic value (e.g., benefits to social image), we posit that the mechanism that physical self-determines consumer behavior differs from that of global self-concepts.

## Theoretical Foundation

### Utilitarian Value Perspective

The utilitarian value perspective suggests that IT users evaluate technology according to the extent to which their goal can be realized with the technology (Bernardi et al., 2019).

In this stream of literature, the theory of reasoned action (TRA) and the theory of planned behavior (TPB) have been widely used to understand users’ intention and behavior related to technology (Cenfetelli, 2004). Following this stream, the technology acceptance model (TAM) posits that perception of technology at the individual level can be used to account for the adoption of technology, such as perception of usefulness, perception of ease of use, and task-technology fit. This logic has been similarly applied to the consumption of wearables. For instance, in the case of smart watch consumption, both perceived usefulness and perceived ease of use (PEOU) have been proven to increase consumers’ adoption intention (Choi and Kim, 2016; Chuah et al., 2016). In research on health-related technology, the perception of usefulness for health (e.g., health management) has been proven to positively influence an individual’s intention to use wearables (Hung and Jen, 2012). It has also been established that patients who are satisfied with the health-related value of mobile health monitoring services (MMSs) are more likely to use MMSs (Xiaofei et al., 2021). In sum, the underlying assumption of utilitarian value perspective is that individuals consciously evaluate the goals. However, recent behavior science indicates that goal-directed behavior can be evoked by pre-existent or unconscious factors (Custers and Aarts, 2010), as noted by Triberti et al. (2016) that traditional perspective cannot fully explain why the perception of value differs among individuals in technology adoption. To assess the pre-existent role of physical self, we incorporate the perception of utilitarian value (health function) as part of our model.

### Symbolic Value Perspective

Although utilitarian value perspective has been widely applied in technology diffusion, some argue that the perception of technology is also related to external variables; in other words, the perception of technology is affected by factors beyond technology (Swanson, 2019). For example, the unified theory of acceptance and use of technology (UTAUT) and technology acceptance 2 (TAM2) theory both propose that non-utilitarian factors involving social influence, i.e., social image and social norms, should be taken into consideration (Venkatesh et al., 2003). Similarly, emotional design emphasizes non-utilitarian factors (e.g., aesthetic, pleasure) in technology usage other than utility factors (Heidig et al., 2015). In contrast with the utilitarian view, symbolic value perspective emphasizes that certain product attributes meet the non-utilitarian demands that involve expressing one’s unique personality or indicating expected social status (Tian et al., 2001; Wilcox et al., 2009). From this perspective, the consumption of counterfeit products, luxury products, and name-brand products are motivated by the symbolic value rather than a specific function or the quality of products (Wilcox et al., 2009; Wolter et al., 2016).

In the context of technology consumption, the symbolic value of digital devices has been proven to enhance purchase intention. For instance, in a study of migrant workers in the emerging market, Huang and Wang (2018) found that name-brand smartphone consumption is driven by consumers’ motivation to associate themselves with people with higher social status, or in other words, the symbolic value contributes to the purchase decision. In another research, perceived self-expressiveness and the need to represent one’s uniqueness as part of one’s social image has been proven to be related to digital consumption (Choi and Kim, 2016). Further supporting this point, the visibility of a smart watch, i.e., being noticed by other people, has been found to increase purchase intention (Chuah et al., 2016). Further, Nieroda et al. (2018) proposed that digital wearables are used by some consumers to communicate idealized social image, i.e., there is symbolic meaning of wearables. Therefore, we have adopted the symbolic value perspective as a portion of our model.

### The Dual Nature of Body Image

In this study, we define physical self as the perception and evaluation of physical self-worth, such as bodily attractiveness and physical conditioning (Fox and Corbin, 1989; Marsh and Redmayne, 1994). In this research, we apply body image as a general measure of physical self. Body image is defined as individual’s evaluation of their body and appearance, and it can be divided into negative and positive body image (PBI) (Cash and Pruzinsky, 2002). Negative body image is defined as perceived inconsistencies between people’s actual and ideal body attributes (Heron and Smyth, 2013). As suggested by Cash et al. (2004), negative body image is related to body image dissatisfaction, as discontent with one’s body image has psychological consequences (i.e., personal distress and adaptive functioning). PBI broadly refers to the acceptance of and appreciation for one’s body, i.e., resistance to social pressure associated with unhealthy and unrealistic body images or emphasizing the physical function of the body rather than appearance (Tylka and Wood-Barcalow, 2015b).

The dual nature of body image refers to the fact that the perception of one’s body and appearance is determined by both physical and social factors (Thompson and Hirschman, 1995; Stowers and Durm, 1996). For instance, individuals may have a negative body image due to being overweight, and their assessment of their weight may be based on an accurate evaluation of their physical condition; other individuals with a healthy weight may negatively evaluate their body image just because their bodies are contrary to media-portrayed ideals (e.g., men need a six-pack in order to be masculine), and this process of evaluation is affected by social norms (Andrew et al., 2014). Therefore, we propose that the dual nature of body image influences behavioral intention via the perception of utilitarian value and symbolic value, and in the context of digital wearable consumption, body image drives both health motivation and self-affirmation.

#### Body Image as a Health Motivation Driver

Health motivation refers to consumers’ goal-directed arousal related to the belief that they should perform preventive actions prior to the emergence of health problems (Moorman, 1990; Moorman and Matulich, 1993). Health motivation has been proven to increase health information searching and health behaviors (Moorman and Matulich, 1993). Empirical research reveals that body image is associated with health promoting and health compromising behaviors, which are driven by health motivations, while the effects of PBI and negative image on health motivation differ.

PBI has been proven to be related to higher health motivation. For instance, PBI has been found to increase health promoting behaviors including sun protection, skin screening and seeking medical suggestions (Andrew et al., 2014). However, individuals with negative body image tend to present lower health motivation. Individuals with negative body image experience more social physique anxiety, so they are less likely to place themselves in situations where others may evaluate their body and appearance, such as gyms and sports teams, which further prevents them from engaging in exercise (Brudzynski and Ebben, 2010). Similarly, negative body image has been found to increase exercise avoidance via embarrassment; in other words, individuals dissatisfied with their bodies are more inclined to avoid health behaviors since they tend to avoid been viewed as unskilled in exercise (More et al., 2019). Therefore, body image (PBI and negative body image) is connected to health-related behaviors.

#### Body Image as a Symbolic Consumption Driver

Body image is socialized; that is, the perception and evaluation of one’s body is dominated by existing cultural ideals, social norms, and moralistic prescriptions (Thompson and Hirschman, 1995), and therefore, body image has been considered as part of physical body-worth, which is related to self-concept (Lowery et al., 2005). For instance, self-esteem has been proven to be associated with body image, and PBI predicts positive self-image or self-esteem, so feeling satisfaction about one’s body and appearance is expected to increase one’s confidence (Thompson and Gray, 1995; Stowers and Durm, 1996).

According to self-affirmation theory, individuals are motivated to maintain the integrity of self, so perceived failures to meet social norms may result in adaptive motivations to defend the integrity of the self (Sherman and Cohen, 2006). Consistent with these conceptualizations, recent research reveals how body image influences consumption behavior. For instance, consumers with poor body image demonstrate more preference for augmented reality (AR) based product presentation than for traditional web-based presentation, and the preference for AR can be explained by the fact that the AR-based product presentation portrays a better body image (Yim and Park, 2019). In other words, the image provided by AR can maintain the socially expected body image. Similarly, women exposed to female models’ images experienced body image threats and insecurity, and they also tend to own more shoes and handbags, since accessories facilitate maintenance of their bodily attractiveness (Boyce et al., 2012). In this view, symbolic consumption is critical to maintain body image regulated by culture. Therefore, body image can drive consumers to make consumption decisions that promote an idealized social image.

#### Utilitarian Value, Symbolic Value and the Dual Nature of Body Image

Smart watches possess utilitarian value through their health monitoring and tracing capabilities, and symbolic value has been represented by the improvement of social image or social status. Under the framework of utilitarian-symbolic value, the dual nature of body image is expected to be related to both utilitarian and symbolic value. For instance, for consumers with PBI, since their health motivation is higher, they may value the health benefits of smart watches; on the other hand, based on the view of body image as a self-affirmation motivation driver, body image may motivate consumers to pay more attention to the appearance of smart watches because they are seeking symbolic value in order to maintain an idealized social image. Thus, past research has ignored the role of body image and failed to investigate the potential link between utilitarian-symbolic value and the dual nature of body image. Given the dual nature of body image, we expect that body image relates to purchase intention via utilitarian and symbolic value.

## Hypotheses

According to the literature discussed above, both utilitarian value (e.g., monitoring, tracing and feedback) and symbolic value (e.g., demonstration of social image) are expected to influence the purchase of wearables. Taking the dual nature of body image into account, we assume that body image influences both utilitarian and symbolic value and further determines purchase intention regarding smart watches.

### Direct Effect of Positive Body Image

Empirical research has proven that negative body image is more likely to motivate consumers to purchase than PBI. For instance, a consumer with a poor evaluation of their own body is more likely to purchase accessories to restore their bodily attractiveness (Boyce et al., 2012). Comparatively, a consumer who is more confident in their body is less likely to buy body-involving products (Rosa et al., 2006), and in a study on cosmetics consumption, PBI failed to predict higher consumption intention (Gillen and Dunaev, 2017). Thus, we propose that:

**H1.Positive body image decreases purchase intention regarding smart watches**

### Mediation Role of Perceived Usefulness for Health

This study focuses on the health-related functions of smart watches, and we redefine perceived usefulness for health as the extent to which a consumer believes that the use of a smart watch will provide health-related benefits, such as health tracing, health management, health monitoring. Since body image is a health motivation driver, and PBI predicts higher health motivation and more health behaviors (Andrew et al., 2014), it is reasonable to assume that consumers with PBI pay more attention to the health-related function of smart watches. Therefore, PBI is related to higher perception of a smart watch’s health functions, or in other words, individuals with PBI are more likely to value the health-related features of digital wearables.

Based on the utilitarian perspective, the user’s rational evaluation of whether a technological innovation can realize the user’s goal is the determinant of technology adoption. In this view, the TAM has been widely applied to investigate utilitarian value related to technology. TAM has been built upon TRA and TPB, and it insists that individuals rationally evaluate the potential profits of technological innovations (Cenfetelli, 2004; Bernardi et al., 2019). The core construct of TAM is perceived usefulness, which is measured in the working context or in organizations. This logic has been applied to health-related technologies, indicating that perceived usefulness for health is an important predictor of a user’s intention to adopt mobile health services and hardware (Guo et al., 2020; Xiaofei et al., 2021). Based on the literature discussed above, body image can be viewed as driving force behind health motivation, and individuals with higher PBI are more inclined to pursue healthy behaviors; therefore, we propose that:

**H2.Positive body image enhances purchase intention by increasing perceived usefulness for health**

### Mediation Role of Value-Expressive and Social-Adjustive Functions

According to functional theories of attitude (FTA), attitudes are not irrational but perform valuable functions, and individuals change or hold their attitudes because these attitudes serve a purpose (Smith et al., 1956; Katz, 1960; Shavitt, 1989). Attitudes can perform functions such as expressing one’s values value-expressive function (VEF) or helping self-presentation social-adjustive function (SAF) (Shavitt, 1989; Wilcox et al., 2009). SAF refers to the social symbolic function of specific products that can facilitate the realization of self-presentation (i.e., displaying images related to wealth and higher social status to others), while VEF refers to the demonstration of individuals’ personal value (i.e., conveying personality to others) through the ownership of products (Wilcox et al., 2009). SAF and VEF have been widely used to account for the functions that attitudes perform in symbolic consumption decisions. For instance, perceived self-expressiveness has been found to have a positive role in smart watch adoption (Choi and Kim, 2016), both SAF and value-expressiveness function predict luxury brand consumption (Schade et al., 2016), and research has revealed the more complicated mediation effect of SAF and value-expressiveness function on counterfeit luxury consumption (Wang et al., 2020). Similarly, we propose that the perception of symbolic value (value-expressive and SAF) predicts the consumption of smart watches.

According to the symbolic value perspective, the presentation of higher social status or favorable social image is an important motivation for consumption decisions. This assumption is in line with earlier research arguing that self-perceptions (e.g., self-esteem or self-image) and body image can be improved via consumption in some circumstances (Sirgy, 1982; Thompson and Hirschman, 1995). Based on the assumption that body image is a driving force of self-affirmation motivation, body image is related to both SAF and value-expressiveness function. Specifically, consumers with PBI are more motivated to purchase body-involving products because they are more interested in maintaining their positive self-concept (Rosa et al., 2006; Merle et al., 2012). In other words, PBI drives consumers to affirm their positive self-concept through consumption. Both SAF and VEF are expected to be influenced by PBI; therefore, we propose that:

**H3.Positive body image enhances purchase intention by increasing value-expressive function**

**H4.Positive body image enhances purchase intention by increasing social-adjustive function**

### Moderating Role of Personal Innovativeness Toward Technology

Compared with other types of personal technology such as smartphones, common consumers are less familiar with digital wearables since digital wearables are cutting-edge technology (Choi and Kim, 2016), and consumers have limited knowledge of the function of the latest technological innovations (Yang, 2005). Therefore, consumers with personal traits such as innovativeness are more likely to be familiar with digital wearables. The concept of personal innovativeness toward technology (PITT) has been developed to identify individuals who tend to adopt the latest information technology innovations earlier than others (Agarwal and Prasad, 1998). Consumers with higher PITT have been found to obtain knowledge regarding a specific product category, so PITT is more than a personality trait: it also drives consumers to seek information about technology products (Varma Citrin et al., 2000).

Following this logic, consumers who are more familiar with digital innovations (e.g., with direct and indirect knowledge of smart watches) are more likely to perceive both the utilitarian and symbolic value of this technology because they pay more attention to technology-related knowledge and information. Higher PITT predicts that customers will assign a higher degree of perceived usefulness (Lu et al., 2005) and relative advantage to a product (Yang et al., 2012), and similarly, consumer innovativeness (measured by the tendency to buy new products) has been found to increase consumers’ continuance intention with regard to smart watch usage (Hong et al., 2017). Therefore, we propose that consumers with higher innovativeness are more likely to perceive both utilitarian and symbolic value:

**H5(a).Personal innovativeness toward technology enhances the purchase intention by strengthening the relationship between positive body image and perceived usefulness for health**
**H5(b).Personal innovativeness toward technology enhances the purchase intention by strengthening the relationship between positive body image and value-expressive function**
**H5(c).Personal innovativeness toward technology enhances the purchase intention by strengthening the relationship between positive body image and social-adjustive function**

## Methodology

### Data Collection

Data was collected via mobile phone from students attending a university in China. Participants were recruited through campus WeChat groups, and samples with answering time less than 60 s were deleted. We collected 369 valid online questionnaires, and as 303 respondents did not have smart watches, we used these 303 samples to perform the PLS-SEM analysis. Of the respondents, 44.88% were female and 55.12% were male, so the distribution of gender was well balanced from the demographic perspective. Also of all respondents, 32.67% were aged 18–21, 43.89% were aged 22–25, 17.82% were aged 26–29 and 5.61% were aged 30 and above, so the distribution of age was consistent with the predominant purchasers of digital wearables. Overall, the selected sample was well balanced. Demographic descriptive statistics are shown in Table 1.

**TABLE 1:** Demographic descriptive statistics (*N* = 303).

Gender	Female (44.88%), Male (55.12%)
Age	Aged 18–21 (32.67%); Aged 22–25 (43.89%); Aged 26–29 (17.82%); Aged 30 and above (5.61%)
Income	500 RMB and below (3.3%); 500–1,000 RMB (6.93%); 1,001–1,500 RMB (21.78%); 1,501–2,000 RMB (22.11%); 2,001–3,000 RMB (25.41%); 3,001–5,000 RMB (12.21%); 5,001–8,000 RMB (2.64%); 8,001 RMB and above (5.61%)
Education	Undergraduate (39.93%); Graduate (59.08%); others (0.99%)

### Analytical Approach

Data analysis was performed using structural equation modeling with partial least squares (PLS-SEM) in SmartPLS 3 3.2.9.. We use the PLS-SEM method for two reasons. First, this study entails multiple mediation and moderation requirements, and PLS has been recommended for complex model estimation. For instance, in models involving multiple mediators, PLS has an advantage because it considers all mediators simultaneously in one model rather than using a piecemeal approach (Hair, 2017). Second, compared to traditional covariance-based structural equation modeling (CB-SEM), PLS-SEM uses weighted composites of indicator variables as proxies, which relaxes the assumptions of CB-SEM based on sum scores. This quality makes it suitable for situations where the theory is less developed (Henseler et al., 2014). Since the theory used in our model was not estimated in prior studies, we used PLS-SEM instead of CB-SEM to introduce new variables and new paths.

### Measures

We used scales validated in previous studies for all constructs (see Supplementary Appendix A). The items were measured using a five-point Likert scale, ranging from 1 (*totally disagree*) to 5 (*totally agree*). PBI items were adopted from Tylka and Wood-Barcalow’s (2015a) original research and translated into Chinese, and they have been proven to have reliability and validity for Chinese respondents (Swami et al., 2016).

PEOU was measured with three items adapted from Davis (1989). Perceived usefulness to health (PUH) was measured with three items adapted from Hung and Jen (2012). VEF was measured with three items and SAF was measured with four items, which were adapted from Wilcox et al. (2009). PITT was measured with three items adapted from Agarwal and Prasad (1998). Purchase intention (PI) was measured with two items developed by Kim and Shin (2015). In terms of control variables, cost (CT) was measured with two items adapted from Shin (2009), and we also included demographic variables, such as gender, age and income, as control variables.

## Results

### Measurement Model

To evaluate the measurement model, we assessed reliability and validity. Table 2 exhibits the factor loadings, Cronbach’s alpha, composite reliability and average variance extracted (AVE) for our constructs. First, most loadings were above the recommended threshold of 0.70, three loadings of PBI were lower than 0.70, including pbi1 (0.545), pbi5 (0.672), and pbi8 (0.695), and one PBI item was removed because its loading was lower than 0.5 (“I am comfortable in my body”). As suggested by Hair (2017), three items with loadings lower than 0.7 were deleted to check the increase of composite reliability, and as the increase was minimal (0.918 vs. 0.916), these three items were not removed. The Cronbach’s Alpha and composite reliability of PBI were 0.897 and 0.916 respectively, indicating that the PBI items were reliable. All values of the Cronbach’s Alpha were above the threshold (0.6), and the composite reliability was higher than the accepted threshold (0.70). These results indicated that the measurement of constructs was reliable.

**TABLE 2:** Loadings, reliability, and convergent validity.

Items	Loadings	Cronbach’s alpha	Composite reliability	AVE
PBI1	0.545	0.897	0.916	0.552
PBI2	0.767			
PBI3	0.797			
PBI4	0.810			
PBI5	0.672			
PBI6	0.813			
PBI7	0.825			
PBI8	0.695			
PBI9	0.720			
PEOU1	0.809	0.794	0.879	0.707
PEOU2	0.866			
PEOU3	0.846			
PUH1	0.900	0.862	0.916	0.784
PUH2	0.873			
PUH3	0.883			
VEF1	0.842	0.757	0.859	0.671
VEF2	0.841			
VEF3	0.773			
SAF1	0.765	0.841	0.894	0.678
SAF2	0.807			
SAF3	0.878			
SAF4	0.841			
PI1	0.905	0.764	0.894	0.809
PI2	0.894			
PITT1	0.820	0.724	0.844	0.646
PITT2	0.713			
PITT3	0.869			
CT1	0.931	0.650	0.842	0.728
CT2	0.767			

Second, the AVE was used to assess the convergent validity, and as shown in Table 1, all AVE values were higher than the threshold (0.50) (Fornell and Larcker, 1981). In addition to evaluating convergent validity, we evaluated discriminant validity using the approach proposed by Fornell and Larcker (1981). According to Fornell and Larcker’s (1981) criterion, to determine the discriminant validity, the square root of each construct’s AVE is expected to be above the coefficient of bivariate correlations with other constructs. According to results of Table 3, discriminant validity of most constructs is established because the square root of each construct’s AVE is higher than the correlation coefficients between all opposing constructs. However, the square root of the AVE of SAF and VEF is close to the correlation coefficients between SAF and VEF (0.823 vs. 0.768; 0.819 vs. 0.768), and SAF explains the variance similarly to VEF; in other words, SAF and VEF are correlated. This is because SAF and VEF are highly related sub-dimensions of the same latent construct (FTAs) (Wilcox et al., 2009). To eliminate the potential collinearity caused by the difference in the way researchers and respondents understood the questionnaire constructs (e.g., for some respondents the concept of “express myself” in VEF may be equivalent to “a symbol of social status”), we follow recommendations in using the variance inflation factor (VIF). The VIF values of SAF and VEF were 2.688 and 2.847, respectively, lower than the threshold of 3.3, indicating no collinearity between constructs (Kock and Lynn, 2012).

**TABLE 3:** Fornell and Larcker’s (1981) criterion.

	CT	PBI	PEOU	PI	PITT	PUH	SAF	VEF
CT	0.853							
PBI	−0.039	0.743						
PEOU	0.111	0.185	0.841					
PI	0.231	0.076	0.425	0.899				
PITT	0.131	0.129	0.296	0.538	0.803			
PUH	0.099	0.278	0.631	0.529	0.333	0.885		
SAF	0.163	0.173	0.102	0.506	0.513	0.283	0.824	
VEF	0.137	0.201	0.178	0.455	0.482	0.419	0.768	0.819

*CT, Cost; PBI, Positive body image; PEOU, Perceived ease of use; PI, Purchase intention; PITT, Personal innovativeness toward technology; PUH, Perceived usefulness for health; SAF, Social-adjustive function; VEF, Value-expressive function.*

### Mediation Effects Test

In a departure from to the traditional classification of full, partial and no mediation suggested by Baron and Kenny (1986) and Zhao et al. (2010) proposed three types of mediation: complementary, competitive and direct-only mediation. To assess the mediation effects, we follow an approach proposed by Zhao et al. (2010).

**FIGURE 1:**
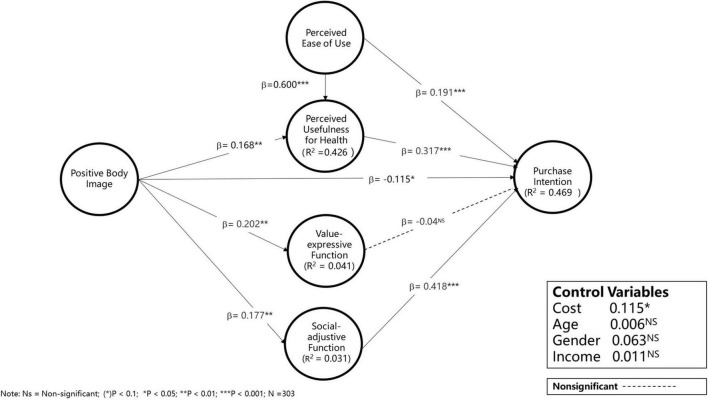
PLS regression results for models without moderation.

We first assess the significance of the indirect effect (β_x→m_ × β_m→y_), and then we further assess the significance of the direct effect (β_x→y_). Complementary partial mediation is determined when direct effect is significant and shares the same direction with indirect effect, or in other words, when β_x→m_ × β_m→y_ × β_x→y_ is positive. In contrast, competitive partial mediation occurs when indirect and direct effect are both significant but move in different directions, i.e., β_x→m_ × β_m→y_ × β_x→y_ is negative. Particularly in competitive mediation, non-significant total effect does not indicate the lack of mediation; for instance, indirect and direct effects of opposite signs may result in the non-significance of the total effect. First, as shown in Table 4, the indirect effect via utilitarian value (PBI→PHU→PI) was significant (β = 0.047, *p* < 0.01), and the indirect effect via social symbol value (PBI→SAF→PI) was significant (β = 0.034, *p* < 0.05). Second, the direct effect (PBI→PI) was also significant but with opposite signs (β = −0.119, *p* < 0.05), indicating a competitive mediation between PBI and PI. Taking all of these effects together, we conclude that PBI enhances purchase intention regarding smart watches via perceived usefulness for health and SAF. The results of the path coefficients are exhibited in Figure 1.

**TABLE 4:** PLS regression results for the mediation model.

	Specific indirect effects	Direct effect	Total indirect effects	Total effect
PBI → PUH → PI	0.047**	−0.116*	0.072**	−0.043^NS^
PBI → VEF → PI	−0.008^NS^	−0.116*	0.072**	−0.043^NS^
PBI → SAF → PI	0.034(*)	−0.116*	0.072**	−0.043^NS^

Ns, Non-significant; *P < 0.1; *P < 0.05; **P < 0.01; ***P < 0.001; N = 303.

### Moderating Effects Test

Moderated mediation indicates that either or both of the paths of independent variables to mediating variables (β_x→m_) and from mediating variables to dependent variables (β_m→y_) vary across levels of the moderator; in other words, indirect effect is contingent on the moderator (Edwards and Lambert, 2007; Preacher et al., 2007).

We used the moderated mediation analysis approaches to test the moderating effects. As shown in Table 5, three distinct models were tested. The first model (Model 1) estimates the moderating effects of PITT on the direct relationship between PBI and PI(β_x→y_), and thus it tests the total effect without mediating effects. The second model (Model 2-1, Model 2-2, Model 2-3) estimates the moderating role of PITT on the first-stage indirect effects, i.e., the effect of PBI on PUH, VEF, and SAF, respectively (β_x→m_). The third model estimates the moderating role of PITT on the second-stage indirect effect, i.e., the simultaneous effects of PUH, VEF, and SAF on PI (β_m→y_) and on the direct effect (β_x→y_).

**TABLE 5:** PLS regression results for the moderated mediation model.

	Model 1. Overall treatment effect (DV:PI, without mediator)	Model 2-1. First-stage mediation (DV: PUH)	Model 2-2. First-stage mediation (DV: VEF)	Model 2-3. First-stage mediation (DV: SAF)	Model 3. Second-stage mediation (DV: PI with mediator)
PBI	−0.039^NS^	0.146**	0.111*	0.079^NS^	−0.100*
PITT	0.446***	0.132*	0.420***	0.471***	0.239***
PBI*PITT	0.088*	0.072^NS^	0.219***	0.184***	0.066^NS^
PUH					0.302***
VEF					−0.053^NS^
SAF					0.314***
PUH*PITT					0.132^NS^
VEF*PITT					−0.108^NS^
SAF*PITT					−0.061^NS^
*Control*					
Age	0.032^NS^				−0.005^NS^
Gender	0.107*				0.053^NS^
Income	−0.007^NS^				−0.000^NS^
CT	0.149**				0.112**
PEOU	0.255***	0.556***			0.127*
Moderated Indirect Effects					
PITT*PBI - > PUH - > PI			0.022^NS^		
PITT*PBI - > VEF - > PI			−0.016^NS^		
PITT*PBI - > SAF - > PI			0.059**		

Ns, Non-significant; *P < 0.1; *P < 0.05; **P < 0.01; ***P < 0.001; N = 303.

**FIGURE 2:**
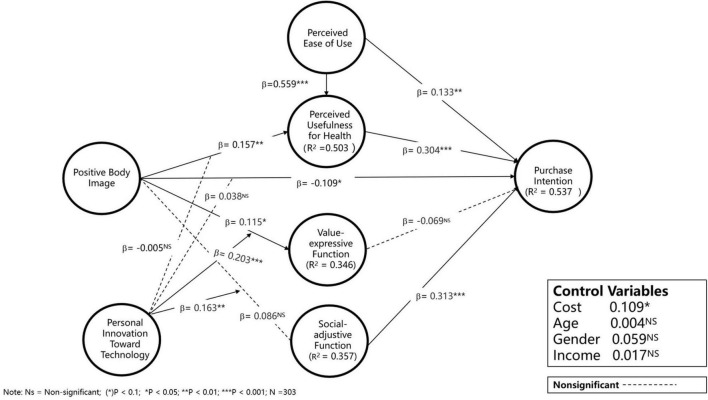
PLS regression results for the models with moderation.

As indicated by results of Model 2-2 and Model 2-3 in Table 5, two of the moderating effects of PITT on the first-stage mediation were significant (β = 0.219, *p* < 0.001 and β = 0.184, *p* < 0.001, respectively), indicating the moderating role of PITT on the effects of PBI on SAF and VEF. First-stage results suggest that individuals with higher PITT perceive more symbolic value associated with smart watches. Turning to the results of second-stage mediation (Model 3), we observe that the coefficients of PUH and SAF were significant (β = 0.302, *p* < 0.001 and β = 0.314, *p* < 0.001). The moderating effect of PITT on the relationship between PBI and SAF in Model 2-2 and on the relationship between SAF and PI were both significant. Taken together, these results indicate that the moderated mediation effect in the indirect effect of PBI on PI via SAF is not zero. This result suggests that the mediation effects partially depend on the extent of PITT (Figure 2).

Additionally, we observe that control variables are non-significant except for cost, and the perception of higher cost predicts higher purchase intention. This finding contrasts with some past research (Kim and Shin, 2015), while some studies argue that high prices in emerging markets are the signal of luxury brands (Sharma et al., 2020). We assume that consumers of digital wearables treat price as a decision reference, or in other words, price is the signal of symbolic value for smart watch consumers.

### Further Explanation of the Dual Nature of Body Image

As discussed above, we assume that body image is a health motivation driver and a symbolic consumption driver, to further prove these assumptions we propose that:

**H6(a).Positive body image enhances health motivation**
**H6(b).Positive body image enhances symbolic consumption motivation**

**FIGURE 3:**
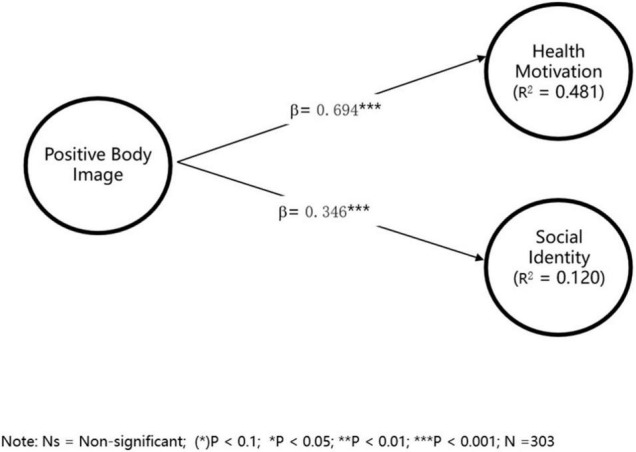
PLS regression results for the dual nature model.

Symbolic consumption motivation was measured with social identity. Social identity refers to a social psychological process in which individual categorize himself as a member of specific groups, such as teams, class, or organizations (Henri and Turner, 1986). In consumption behaviors, consumers purchase specific products to signal favorable social identity, especially for conspicuous products with symbolic value (Wilcox et al., 2009). In this research, we used three items adopted from Moorman (1990) to measure health motivation, and social identity was measured with three items from Huang and Wang (2018), the reliability and validity meet the threshold as in section “Methodology” (see Supplementary Appendix B). Using the same sample as in section “Methodology”, we conclude that H6 (a) and H6 (b) are supported, and PBI has a stronger relationship with health motivation compared with social identity (see Figure 3).

## Discussion

### Key Findings

Drawing on the TAM and on FTA, we find that PBI influences intention to purchase via the perception of technology value and symbolic value, thus establishing the dual nature of body image. First, individuals with a positive evaluation of their own body and appearance (PBI) do not exhibit more purchase intention regarding smart watches, while negative body image predicts a higher degree of purchase intention. Second, PBI increases purchase intention via the perception of the utilitarian value, i.e., the health-related value, of smart watches. Among individuals who evaluate their body image positively, respondents present a higher perception of smart watches’ health promoting functions. Third, PBI increases purchase intention via symbolic value. Specifically, individuals with a more PBI perceive more SAF (e.g., that smart watches gain the owner higher social status and more visibility), which further increases purchase intention.

Additionally, we have proven that PITT moderates the mediating role of SAF. For a summary of the main conclusions (please see Table 6). These findings have significant implications regarding digital wearables, as outlined below.

**TABLE 6:** Summary of results.

Hypotheses	Results
H1. Positive body image decreases purchase intention regarding smart watches	Supported
H2. Positive body image enhances purchase intention by increasing perceived usefulness for health	Supported
H3. Positive body image enhances purchase intention by increasing value-expressive function	Unsupported
H4. Positive body image enhances purchase intention by increasing social-adjustive function	Supported
H5 (a). Personal innovativeness toward technology strengthens the relationship between positive body image and perceived usefulness for health	Unsupported
H5 (b). Personal innovativeness toward technology strengthens the relationship between positive body image and value-expressive function	Unsupported
H5 (c). Personal innovativeness toward technology strengthens the relationship between positive body image and social-adjustive function	Supported
H6 (a). Positive body image enhances health motivation	Supported
H6 (b). Positive body image enhances symbolic consumption motivation	Supported

### Theoretical Contribution

According to our knowledge, this study is the first work to assess the role of physical self in consumption behavior. Self-concepts are individuals’ evaluation toward themselves such as self-esteem (global perceptions of one’s worth) (Harter and Leahy, 2001), compared to global self-concepts, body is considered as domain-specific self-concepts (e.g., body size satisfaction and appearance esteem). Although body image has been widely applied in research into health management topics such as eating disorders and obesity (Cash and Pruzinsky, 2002), the relationship between body image and consumption has remains unknown. For instance, researchers do not agree on how body image (either negative or positive) affects purchase intention (Rosa et al., 2006), this research establishes that without considering the dual nature of body image (indirect effect), such mechanisms cannot be revealed. Therefore, in the view of physical self, we reveal the mechanism driving the effect of body image by introducing the dual nature of body image and demonstrating that body image is a health motivation driver (e.g., influencing the perception of utilitarian value) and also a self-affirmation motivation driver (e.g., influencing the perception of symbolic value).

In other words, our research provides a theoretical framework to understand the role of physical self-concepts in consumption. Self-concepts define who we are and become a driving force to influence consumption decision, however, self-concepts is also multi-dimensional (Belk, 1988, 2013), the investigation into body-involving products (e.g., clothes, sports, and wearables) requires the introduction of body dimension of self. Furthermore, our findings have implications for research based on self-concepts that self-concepts (global or domain-specific) should be recognized as antecedent variables, and their significance in motivating consumers’ decisions should be given more consideration.

### Practical Implications

Although designers and researchers have cumulative knowledge of marketing strategy for digital wearables, our results have two critical implications for design and marketing.

First, the hybrid nature of digital wearables does not necessarily relate to hybrid marketing strategy, i.e., advertisements do not need to emphasize devices’ utilitarian and symbolic value simultaneously. Our results suggest that for consumers with PBI, the health-related function will have direct and significant convincing power, while the realization of symbolic value depends on the knowledge on digital products. More importantly, the health-related functions can attract consumers’ attention. For instance, most mainstream design tends to transform smart watches into mini smartphones (e.g., they have payment, messaging and notification features), and the overlap between smartphones and smart watches increases the burden of making a purchase decision.

Second, designers wish to copy the experience of traditional luxury wearables; convincing consumers that the ownership of smart watches can have symbolic value has already become a prevalent strategy. However, our results indicate that consumers’ past experience may not fully apply to digital wearables, i.e., the perception of symbolic value is affected by the perception of body image. Specifically, consumers with positive attitudes toward their own body and appearance tend to focus on the symbolic value, while negative body image decreases the effect of symbolic value. Additionally, consumers’ knowledge of digital wearables may limit their perception of symbolic value. Compared with more traditional and prevalent luxury wearables, consumers are less familiar with digital wearables. We therefore posit that strategies that are successful for luxury wearables may not work on all potential consumers of digital wearables. For example, given Apple’s advantage in brand premium, Huawei and Xiaomi, two of Apple’s major competitors in China, adopted the differentiation strategy by releasing cheaper smart bracelets from 2016 to 2018. These products have a smaller screen but similar health functions to the Apple Watch.

### Limitations and Future Studies

Although the model proposed in this study provides a more comprehensive perspective from which to understand a consumer’s intention to purchase a smart watch, our findings have several limitations. First, our samples are limited to college students, and due to its exploratory purpose, our study uses a convenient sampling method. Despite the fact that young students are a major portion of digital wearable consumers, older respondents (e.g., age 30–40) with different careers should be involved in future research, since consumers with various socioeconomic statuses (SES) and at different life stages may hold different attitudes about their physical self, and the dual nature of physical self may be influenced by these factors. Second, although we tested the theoretical validity of physical self in the context of digital product consumption, a deeper exploration of physical self in wearable consumption is needed. Since studies considering the body-involving features of digital wearables were rare in the past, the theoretical framework applied in this article requires more solid theoretical discussions (e.g., the links to identity, self-image, and self-efficiency). Third, the diffusion of technology involves multiple stages, such as adoption, acceptance, routinization, exploration and infusion, while this research only focuses on purchase intention, we suggest future research to investigate other related topics, for instance, using samples with actual purchase behavior.

## Data Availability Statement

The original contributions presented in the study are included in the article/Supplementary Material, further inquiries can be directed to the corresponding author/s.

## Author Contributions

TW developed the theoretical framework and performed the online survey and wrote the manuscript with support from SL. YS suggested the selection of measurements and checked the manuscript. All authors discussed the results and contributed to the final manuscript.

## Conflict of Interest

The authors declare that the research was conducted in the absence of any commercial or financial relationships that could be construed as a potential conflict of interest.

## Publisher’s Note

All claims expressed in this article are solely those of the authors and do not necessarily represent those of their affiliated organizations, or those of the publisher, the editors and the reviewers. Any product that may be evaluated in this article, or claim that may be made by its manufacturer, is not guaranteed or endorsed by the publisher.

## Footnotes

1
https://www.idc.com/getdoc.jsp?containerId=prUS46885820
