# Supplementary material for: Physical Self Matters: How the Dual Nature of Body Image Influences Smart Watch Purchase Intention
Source: Front Psychol. 2022 Mar 25;13:846491. doi: 10.3389/fpsyg.2022.846491 (PMC8992001; doi:10.3389/fpsyg.2022.846491)
Supplement: Supplementary file 1 [file Table_1.docx]

Appendix A

| Construct | In English |
| --- | --- |
| Positive body image (PBI) | **Source: Tylka & Wood-Barcalow (2015a)**  I respect my body（PBI1）  I feel good about my body（PBI2）  I feel that my body has at least some good qualities（PBI3）  I take a positive attitude towards my body（PBI4）  I am attentive to my body’s needs（PBI5）  I feel love for my body（PBI6）  I appreciate the different and unique characteristics of my body（PBI7）  My behavior reveals my positive attitude toward my body; for example, I hold my head high and smile（PBI8）  I feel like I am beautiful even if I am different from media images of attractive people (e.g., models, actresses/actors) (PBI9) |
| Value-expressive function (VEF) | **Source: Wilcox et al. (2009)**  Smart watches help me communicate my self-identity (VEF1)  Smart watches help me define myself (VEF2)  Smart watches help me express myself (VEF3) |
| Social-adjustive function（SAF） | **Source: Wilcox et al. (2009)**  Smart watches are a symbol of social status(SAF1)  Smart watches help me fit into important social situations(SAF2)  I like to be seen wearing Smart watches (SAF3)  I enjoy it when people know I am wearing a Smart watch (SAF4) |
| Perceived usefulness to health (PUH) | **Source:** Hung & Jen (2012)  Using a smart watch will let me know my body condition in real time（PUH1）  Using a smart watch will enhance the effectiveness of tracing and recording my body condition （PUH2）  Using a smart watch will be useful in managing my health（PUH3） |
| Perceived ease of use (PEOU) | **Source: Davis (1989)**  Learning to operate smart watches would be easy for me (PEOU1)  I would find it easy to get smart watches to do what I want it to do (PEOU2)  I would find smart watches easy to use(PEOU3) |
| Cost (CT) | **Source: Shin (2009)**  Smart watches were expensive (CT1)  Purchasing a smart watch was a burden to me (CT2) |
| Purchase intention (PI) | **Source: Kim shin (2015)**  I intend to buy a smart watch in the near future (PI1)  If I had the financial resources to afford a smart watch, I would buy one (PI2) |
| Personal innovativeness toward technology (PITT) | **Source: Agarwal & Prasad (1998)**  If I heard about a new information technology, I would look for ways to experiment with it (PITT1)  Among my peers, I am usually the first to try out new information technologies (PITT2)  In general I am hesitant to try out new information technologies (PITT3) |
| Health motivation (HM) | **Source: Source: (Moorman & Matulich, 1993)**  I try to prevent health problems before I feel any symptoms (HM1)  I am concerned about health hazards and try to take action to prevent them (HM2)  I try to protect myself against health hazards I hear about (HM3) |
| Social identity (SI) | **Source: (Huang & Wang, 2018)**  I would consider my social identity when making purchase decision(SI1)  How to spend should match one's social identity(SI2)  Lifestyle and consumption should fit one's social identity(SI3) |

Appendix B

| Items | Loadings | Cronbach’s  Alpha | Composite reliability | AVE |
| --- | --- | --- | --- | --- |
| PBI1 | 0.650 | 0.897 | 0.915 | 0.547 |
| PBI2 | 0.739 |  |  |  |
| PBI3 | 0.790 |  |  |  |
| PBI4 | 0.777 |  |  |  |
| PBI5 | 0.737 |  |  |  |
| PBI6 | 0.796 |  |  |  |
| PBI7 | 0.790 |  |  |  |
| PBI8 | 0.693 |  |  |  |
| PBI9 | 0.670 |  |  |  |
| HM1 | 0.830 | 0.775 | 0.870 | 0.690 |
| HM2 | 0.850 |  |  |  |
| HM3 | 0.812 |  |  |  |
| SI1 | 0.778 | 0.749 | 0.851 | 0.656 |
| SI2 | 0.794 |  |  |  |
| SI3 | 0.856 |  |  |  |

|  | HM | PBI | SI |
| --- | --- | --- | --- |
| HM | 0.831 |  |  |
| PBI | 0.694 | 0.740 |  |
| SI | 0.338 | 0.346 | 0.810 |
